# The integral spliceosomal component CWC15 is required for development in Arabidopsis

**DOI:** 10.1038/s41598-020-70324-3

**Published:** 2020-08-07

**Authors:** Daniel Slane, Cameron H. Lee, Martina Kolb, Craig Dent, Yingjing Miao, Mirita Franz-Wachtel, Steffen Lau, Boris Maček, Sureshkumar Balasubramanian, Martin Bayer, Gerd Jürgens

**Affiliations:** 1 Max Planck Institute for Developmental Biology, Cell Biology, 72076 Tübingen, Germany; 2 Howard Hughes Medical Institute, Division of Basic Sciences, Fred Hutchinson Cancer Research Center, Seattle, WA 98109 USA; 3 School of Biological Sciences, Monash University, Clayton Campus, Clayton, VIC 3800 Australia; 4 Proteome Center Tübingen, University of Tübingen, Auf der Morgenstelle 15, 72076 Tübingen, Germany

**Keywords:** Embryogenesis, Germline development, Developmental biology, Development, Gene expression, Gene regulation, Genetics, Transcriptomics, Proteomics, Alternative splicing, RNA splicing

## Abstract

Efficient mRNA splicing is a prerequisite for protein biosynthesis and the eukaryotic splicing machinery is evolutionarily conserved among species of various phyla. At its catalytic core resides the activated splicing complex Bact consisting of the three small nuclear ribonucleoprotein complexes (snRNPs) U2, U5 and U6 and the so-called NineTeen complex (NTC) which is important for spliceosomal activation. CWC15 is an integral part of the NTC in humans and it is associated with the NTC in other species. Here we show the ubiquitous expression and developmental importance of the Arabidopsis ortholog of yeast *CWC15*. CWC15 associates with core components of the Arabidopsis NTC and its loss leads to inefficient splicing. Consistent with the central role of CWC15 in RNA splicing, *cwc15* mutants are embryo lethal and additionally display strong defects in the female haploid phase. Interestingly, the haploid male gametophyte or pollen in Arabidopsis, on the other hand, can cope without functional *CWC15*, suggesting that developing pollen might be more tolerant to CWC15-mediated defects in splicing than either embryo or female gametophyte.

## Introduction

Angiosperms are the predominant group of land plants. A hallmark of this dominance in the course of evolution is the establishment of a reduced haploid phase (called gametophyte) in the life cycle of flowering plants. In free-living gametophytes of mosses, the gametophytic generation forms independently recognizable plants that can even be the dominant structure. In contrast, the gametophyte of flowering plants is reduced to a small dependent structure with an almost minimal number of cells and a short lifetime^1^. In Arabidopsis, the female gametophyte is deeply embedded in sporophytic tissue, whereas the male gametophyte or pollen has to be released from the sporophytic anther tissue for pollination to occur. Upon successful pollen-stigma interaction, the pollen tube grows inside the transmitting tract towards the ovule. Recent research has revealed that several signaling molecules including peptides play a role in the guidance of the pollen tube, attraction by the female gametophyte and burst of the pollen tube tip within one of the two synergid cells. If any of the aforementioned processes is disrupted, fertilization does not take place. Several mutants with disruptions in these processes have been isolated in the past^1–3^. Upon successful fertilization, the Arabidopsis zygote initiates a precise developmental program, which results in a heart-shaped embryo already comprising all major seedling organs: two primary leaves or cotyledons, a shoot meristem, a hypocotyl, and a primary root with a root meristem^4^. This invariant embryo patterning and development is impaired in mutants defective for various cellular response pathways e.g. responses to phytohormones, small RNA pathways, vesicular trafficking, cytoskeletal structure, and cell cycle control^5–9^. Furthermore, mutations in genes that are components of the RNA splicing machinery (spliceosome) affect gametophyte function and embryogenesis. For example, mutations such as *gfa1/clotho*, *rtf2*, *sus2/prp8*, or *bud13* cause severely reduced transmission of the mutant alleles via the female gametophyte and cause embryo lethality in Arabidopsis^10–14^.

The pivotal step of splicing—intron removal—constitutes two *trans*-esterification reactions, mediated by the spliceosome, a dynamic protein complex containing more than 100 proteins and 5 small nuclear ribonucleoprotein particles (snRNP)^15^, which is likely highly conserved among eukaryotes. The snRNPs consecutively interact with the pre-mRNA. First, U1 snRNP and U2 snRNP interact with the splice and the branch site, respectively. Then U4/U6-U5 snRNPs and the PRP19-CDCL5 complex (so-called NineTeen complex [NTC] in yeast) associate, thereby forming the pre-catalytic spliceosome^16^. After the dissociation of U4 snRNP, the Prp19 complex stabilizes the interaction of U5 snRNP and U6 snRNP with the spliceosome^17,18^. Recent studies using cryo-EM uncovered detailed spliceosomal structures during various steps of mRNA splicing. The NTC/PRP19 complex is highly conserved between yeast and human and contains six and seven core proteins, respectively^19–22^. Important for the function of the active spliceosome are also the so-called NTC-related (NTR) proteins, of which CWC15 is a member.

The developmental importance of Cwc15 was shown in yeast as a loss of function of Cwc15 confers lethality in *Schizosaccharomyces pombe* and it is synthetically lethal with *prp19-1* in *Saccharomyces cerevisiae*^23^. Furthermore, in *S. cerevisiae* it was shown that the core spliceosome components are not equally important for all pre-mRNAs, perhaps explaining why in Arabidopsis the absence of several components might affect tissues differently^24^. Regarding multicellular eukaryotes, *CWC15* was suggested to be important for bovine embryo development^25^. In *Arabidopsis thaliana,* CWC15 was not found in a proteomic approach as a member of the NTC^26^. Many genes coding for components of the core splicing machinery are duplicated in Arabidopsis although mutations in single-copy genes frequently result in gametophytic cell death^11,26^. Interestingly, the phenotypic consequences of mutations in spliceosomal genes are different between female and male gametophytes. Mutations in CLOTHO, which is a homolog of the yeast U5-associated Snu114, and ATROPOS, whose homolog has a demonstrated role in U2 assembly^27^, result in defective female gametophytes, whereas male transmission is less severely affected^11^.

In this work, we address the importance of the predicted splicing factor CWC15 in the model plant *Arabidopsis thaliana*. Our results show that CWC15 is associated with homologs of core yeast and human spliceosome components. Furthermore, CWC15 is essential for plant development including embryo development as splicing is affected on a whole-genome level. CWC15 also plays some role in the female gametophyte, however, pollen development proceeds normally in the absence of CWC15.

## Results

### *CWC15* encodes a highly conserved splicing factor with ubiquitous expression

CWC15 was initially described as a spliceosome-associated protein in yeast and human cells. Subsequent cryoEM studies placed it within the core machinery of the spliceosome^15,28^. Our thorough phylogenetic analysis revealed the evolutionary conservation of CWC15 across all eukaryotes (Supplementary Fig. 1). CWC15 protein appears to have diverged between plants and animals, with specific amino acid sequences distinguishing the clades (Supplementary Fig. 1A and B). Nevertheless, major domains especially in the N- and C-terminal parts of the protein homologs appear to be conserved, which suggests the general importance of CWC15 during splicing in all eukaryotes.

**Figure 1:**
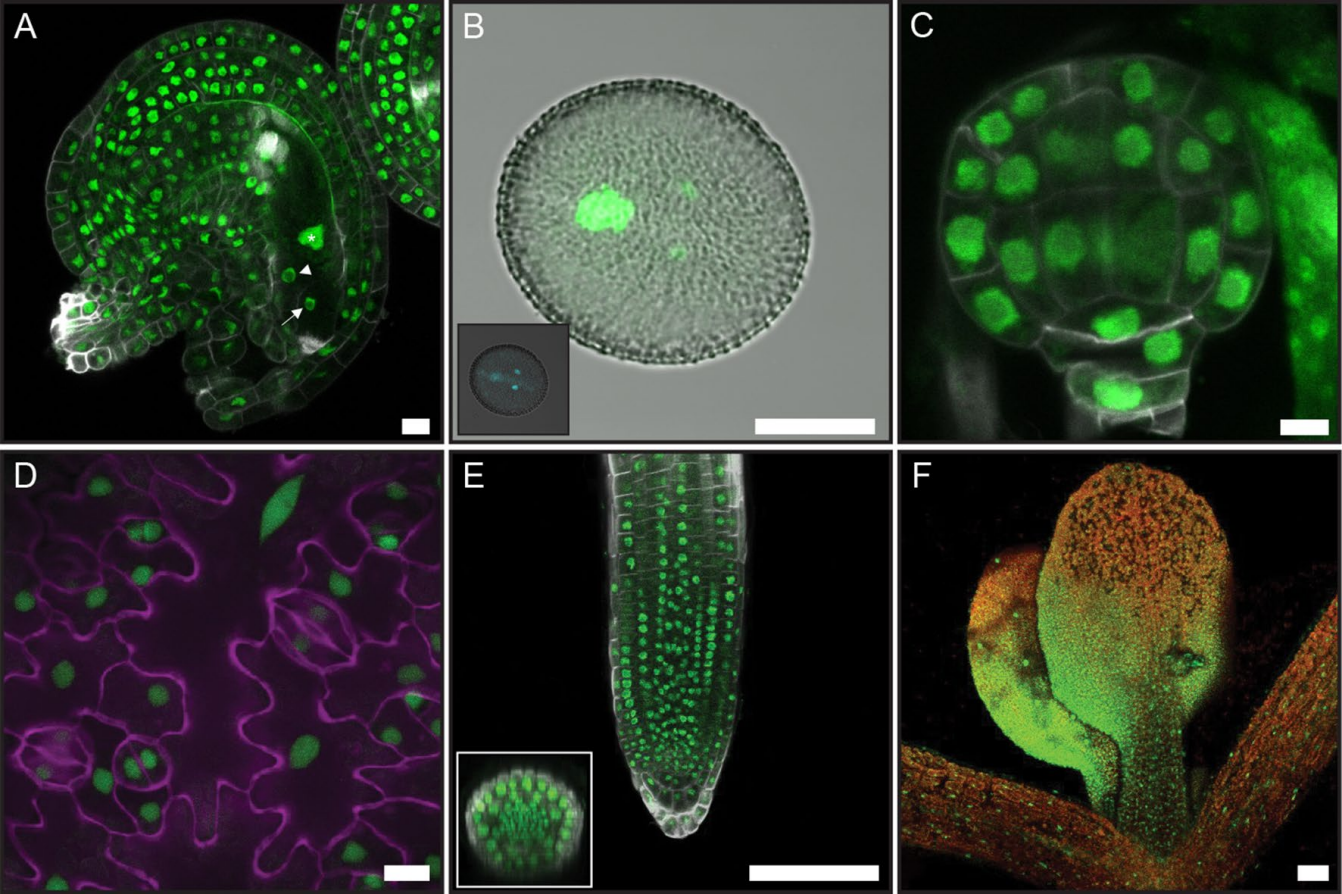
Expression pattern of genomic construct *gCWC15-3xGFP* during plant development. *CWC15* expression pattern monitored with a genomic fusion to GFP is visible in all nuclei of gametophytes, embryo, and seedling. (**A**) Mature female gametophyte with nuclear GFP signal in all tissue types including central cell (asterisk), synergid (arrowhead), and egg cell (arrow). (**B**) Both vegetative and generative nuclei show CWC15-3xGFP signals in mature pollen grain. Inset: DAPI-stained nuclei in the mature pollen grain. (**C**) CWC15-3xGFP is present in all nuclei at globular embryo stage. (**D**) *gCWC15-3xGFP* expression in epidermal cells. The image is a maximum projection of z-stacks across abaxial cotyledon epidermal cells. Nuclear-localized CWC15-3xGFP is shown in green, cell outlines are stained with propidium iodide (magenta). (**E**) *gCWC15-3xGFP* expression in seedling root. Nuclear-localized CWC15-3xGFP is shown in green, cell outlines are stained with Renaissance SR2200 dye (grey). Transverse root section is shown as inset. (**F**) *gCWC15-3xGFP* expression in seedling shoot. Nuclear-localized CWC15-3xGFP in primary leaves of a 7-day-old seedling is shown in green, autofluorescence is shown in red. Scale bar: (**A**–**D**) 5 μm, (**E**,**F**) 100 μm.

To assess *CWC15* expression, we expressed a translational fusion of 3xGFP to a genomic rescue construct. The GFP signal was exclusively nuclear which is consistent with the potential role of CWC15 as a splicing factor. The fusion protein CWC15-3xGFP was ubiquitously expressed in all gametophyte, embryo, and seedling tissues and here too localized to the nucleus (Fig. 1). The integuments and all cells of the mature, unfertilized embryo sac showed GFP fluorescence, including central cell, synergids, and the egg cell (Fig. 1A). Likewise, the male gametophyte was marked by nuclear fluorescence during all developmental stages from unicellular microspore to tricellular, mature pollen (Fig. 1B, Supplementary Fig. 2A–C). Also, all cells of the embryo at the early globular (Supplementary Fig. 2D–F), globular and triangular (Fig. 1C, Supplementary Fig. 2G), late-heart or torpedo and bent-cotyledon stages (Supplementary Fig. 2H–J) showed clear nuclear fluorescent signals. We were also able to detect nuclear fluorescent signals in all cells of seedling tissues such as the cotyledon epidermis with stomata and pavement cells (Fig. 1D, Supplementary Fig. 2K), the primary root with all radially organized cell layers (Fig. 1E), the hypocotyl and the first rosette leaves including trichomes (Fig. 1F, Supplementary Fig. 2L). In summary, *CWC15* encodes a ubiquitously expressed, nuclear-localized protein.

### CWC15 is closely associated with the Arabidopsis NTC

In yeast and human, CWC15 is an integral part of the core spliceosome^20,21^. To assess whether CWC15 is a component of the spliceosome in Arabidopsis, we performed immunoprecipitation experiments with GFP-tagged CWC15 and analyzed the precipitates by LC–MS/MS. As controls, we used GFP-tagged IMPORTIN-ALPHA 6 (IMPα6) and transcription factor AUXIN RESPONSE FACTOR 5 (ARF5) and carried out immunoprecipitation followed by liquid chromatography–mass spectrometry (LC–MS). Both IMPα6 and ARF5 are also localized to the nucleus, but functionally distinct from CWC15^29,30^. We looked for peptides that were specifically enriched in the CWC15 but absent in the two other immuno-precipitates. The most abundant peptides recovered were Arabidopsis counterparts of the human Prp19 complex (NTC), U5 snRNP, and NTC-related proteins (NTR) (Table 1). The majority of these mapped to Arabidopsis homologs of human spliceosomal proteins of the NTC such as Cdc5 and Prp19, two proteins that were well described in their function for NTC-related spliceosomal activation^31^. In addition to CWC15 itself (Ad-002 in human spliceosome), we found a homolog for the human NTC-related (NTR) protein Aquarius, which like CWC15 is required for embryo viability in Arabidopsis (*EMB2765*)^32^. Adding peptides with lower counts to our analysis, we detected a majority of all components of the U5 snRNP, NTC, NTR, and associated splicing factors (Supplementary Table 2) that were recently described in a multitude of structural cryo-EM reports for yeast and human spliceosomes^15,28^. These results suggest that CWC15 is potentially part of the NineTeen complex, which has an important general role in splicing in *Arabidopsis thaliana*^26,33^.

**Table 1:** CWC15-associated proteins identified by mass spectrometry.

*Homo sapiens*	*Arabidopsis thaliana*	AGI locus	Peptides
*U5 snRNP*			
Prp8	PRP8B	*AT4G38780*	23
Snu114	GFA1/MEE5/CLO	*AT1G06220*	14
*NTC*			
Cdc5	CDC5/MAC1	*AT1G09770*	18
Prp19	MAC3A	*AT1G04510*	12
	MAC3B	*AT2G33340*	12
Syf1	MAC9	*AT5G28740*	14
Syf3	MAC10	*AT5G41770*	13
HSP73	HSP70	*AT3G09440*	21
*NTR*			
Ad-002	CWC15	*AT3G13200*	26
Aquarius	EMB2765	*AT2G38770*	12

Recovered unique peptides were compared to MS data for two other nuclear-localized proteins (IMPα6 and ARF5) and only peptides that were not present in the other two data sets are listed. Only loci with more than 10 unique peptide counts are depicted.

### Down-regulation of *CWC15* causes developmental defects

**Figure 2:**
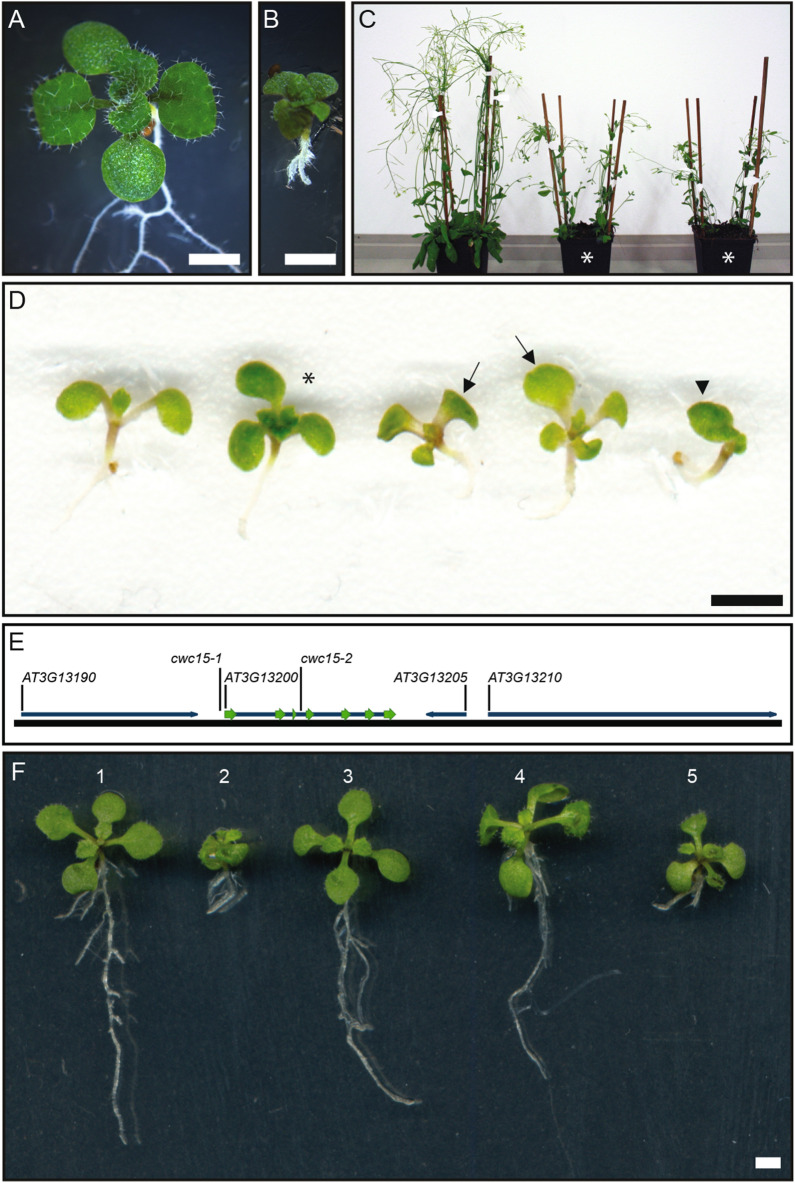
Mis-regulation of *CWC15* is causative for embryo and seedling phenotypes. (**A**,**B**) Normal wild-type (**A**) and *cwc15-1* mutant seedling (**B**) with short primary root and three cotyledons. (**C**) Adult *cwc15-1* mutant plants (marked by asterisks) grow smaller compared to wild type. (**D**) Range of *cwc15-1* mutant seedling phenotypes with wild-type seedling on the left. Asterisk marks tricot seedling with normal root, arrows point to seedlings with asymmetrically positioned cotyledons, and arrowhead marks seedling with only one fully developed cotyledon. (**E**) The insertion site of the transgene is in the vicinity of 4 gene loci, among them *CWC15* (*At3g13200*). Mutant alleles with corresponding insertion sites are depicted as *cwc15-1* (promoter) and *cwc15-2* (SALK insertion line, 3rd intron). Exons are shown as green arrows. Image of gene loci was exported from CLC Genomics Workbench software version 10.1.1 (https://digitalinsights.qiagen.com/products-overview/discovery-insights-portfolio/analysis-and-visualization/qiagen-clc-genomics-workbench/). (**F**) *cwc15-1* rescue lines (at least 6 independent transgenic lines tested for each construct) with seedling phenotypes from left to right: (1) Wild type, (2) *cwc15-1* mutant, (3) genomic rescue, (4) rescue with constitutive expression from ribosomal *RPS5A* promoter, (5) no rescue if expressed from putative promoter of gene *At3g10100* which is only active during early embryogenesis. Scale bar: 2 mm.

In an enhancer trap screen, we identified a T-DNA insertional mutant that displayed several phenotypic features reminiscent of auxin-related defects. Compared to wild type, mutant seedlings and adult plants were strongly reduced in size (Fig. 2A–C). Adult plants were fertile despite stunted growth when compared to Col-0 wild-type plants (Fig. 2C). Mutant seedlings displayed stunted primary roots (Fig. 2B,D) and cotyledon defects ranging from monocots or asymmetrically positioned cotyledons to seedlings with three cotyledons (Fig. 2D). Phenotypic defects were visible in all offspring seedlings from homozygous mother plants when grown on agar plates while heterozygous plants did not show any obvious defects.

To determine the genomic insertion site of the transgene that caused the mutant phenotype, we sequenced the entire genome and aligned DNA sequencing reads both to the T-DNA used and the Arabidopsis genome as was previously described^34^. The insertion was located on the upper arm of chromosome 3 directly upstream of the genomic locus *CWC15/AT3G13200* (Fig. 2E). We tested the possible effects of the insertion on the expression of genes near the insertion site through semi-quantitative (sq) RT-PCR (Supplementary Fig. 3). We found that *CWC15/AT3G13200* was downregulated and confirmed the strong down-regulation also by quantitative (q) RT-PCR (Supplementary Fig. 4A). Among the other genes flanking the insertion site, we observed additional bands for *AT3G13190* and an up-regulation of *AT3G13210*. *AT3G13205* is a predicted pseudogene. Both *CWC15/AT3G13200* and *AT3G13210* code for putative splicing factors and the additional transcripts observed for *AT3G13190* suggested possible splicing defects in the mutant. Since multiple homozygous T-DNA insertion lines located in exons are available for *AT3G13210* and therefore its absence is not deleterious for development, we focused on *CWC15/AT3G13200*, the homolog of the yeast/human splicing factor *Cwc15/AD-002*. We termed the mutant therefore *cwc15-1*. A genomic construct expressing the *CWC15* gene from about 1 kb of the upstream sequence was able to fully complement the mutant seedling phenotypes (Fig. 2F). The same was observed when a strong ribosomal promoter *RPS5A* drove expression of *CWC15*. Interestingly, expression from a promoter only active during early embryogenesis^35^ did not rescue the observed seedling defects, suggesting that continued protein activity during later embryo and seedling development might be necessary (Fig. 2F).

**Figure 3:**
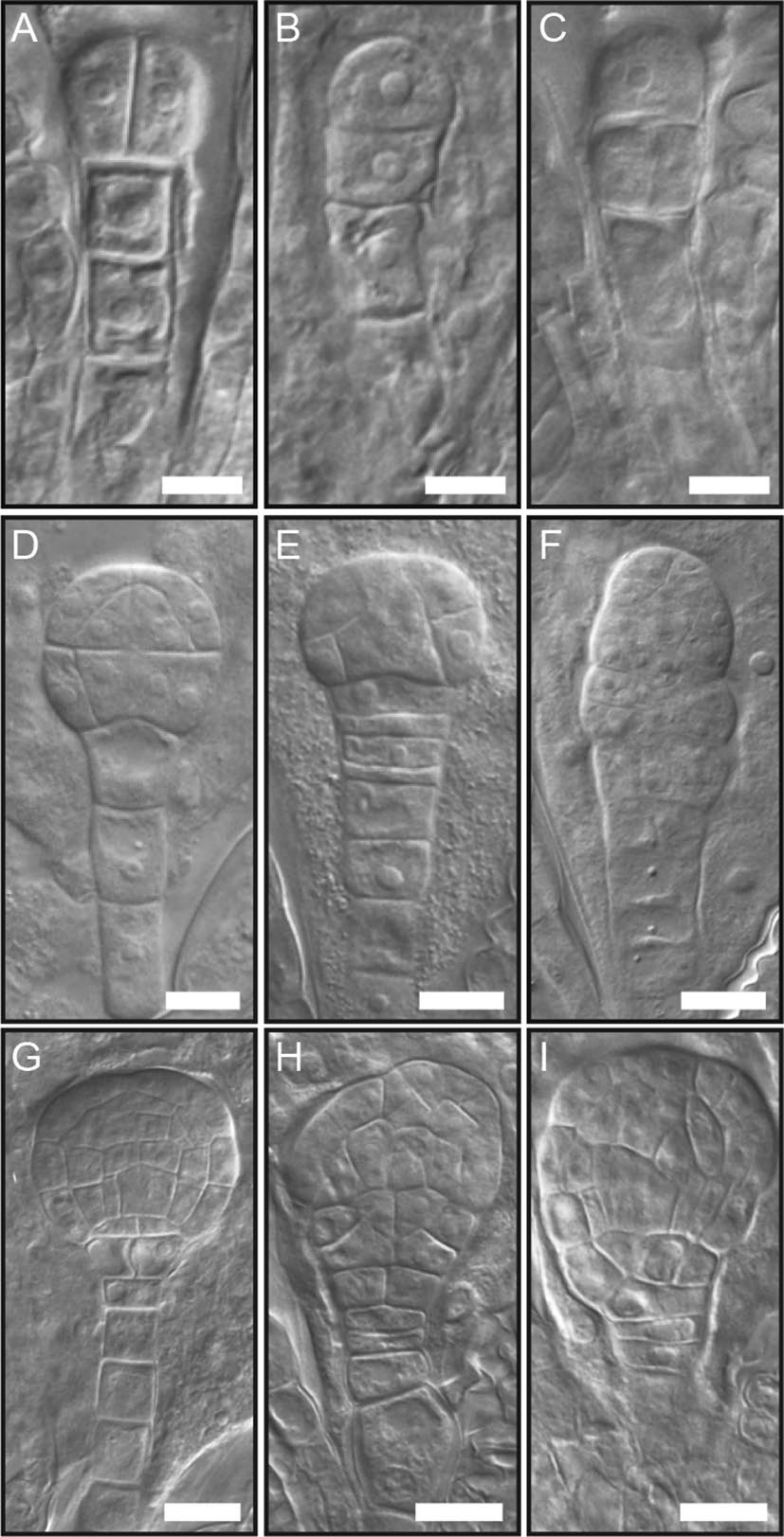
Embryo phenotypes at early stages of development. (**A**–**I**) Corresponding embryos are shown for 2-cell (**A**–**C**), 16-cell (**D**–**F**), and globular stages (**G**–**I**). Wild-type embryos are depicted in (**A**,**D**,**G**) and mutant embryos in (**B**,**C**,**E**,**F**,**H**,**I**). Scale bar: 10 µm.

To elucidate the earliest deviation in development, we analyzed embryos from 2-cell to mid-globular stages comparing wild type to the *cwc15-1* mutant. In general, mutant embryos showed a variety of strongly pleiotropic embryo defects when compared to wild type. In Col-0 embryos, the division plane of the apical daughter cell of the zygote is vertical (Fig. 3A). In contrast, mutant embryos often showed a horizontal division plane (Fig. 3B). Also, we observed frequent erroneous divisions in the basal cell lineage of the embryo (Fig. 3C). These phenotypes are for example reminiscent of embryo phenotypes observed in *yda* or *wrky2* mutants^36,37^. When the wild-type embryos were at the 16-cell stage (Fig. 3D), mutant embryos displayed altered division planes, to varying degrees exhibiting raspberry-like phenotypes^38^ (Fig. 3E,F). At mid-globular stage (Fig. 3G)—a time point when an asymmetric division of the so-called hypophysis establishes the root—apical and basal domains appeared strongly misshapen, resembling *fass* mutant phenotypes^39^ (Fig. 3H,I). In conclusion, mutant embryos displayed a range of phenotypic alterations, which are similar to already described embryo mutants and this suggested that there is potentially mis-regulation of multiple genes during early embryogenesis in the *cwc15-1* mutant.

### CWC15 is important for efficient splicing

To determine the extent of potential splicing defects in *cwc15* mutants, we performed RNA sequencing on total RNA extracted from tissues representing the early and late stages of development. First, we analyzed RNA from wild-type and hypomorphic *cwc15-1* seedlings, where we observed a clear phenotypic difference. Second, we did RNA-seq profiling on total RNA extracted from mature pollen tissue from wild-type and *cwc15-1* mutants. To assess the comparability of biological replicates we used principal component analysis. Tissue/developmental difference was the major component of variation (accounting for ~ 81%) in expression levels (Supplementary Figure 5A).

To analyze whether specific splice sites across the genome are affected in *cwc15-1* mutants, we utilized SpliSER^40^, which enables the quantification of splicing at the level of individual splice sites. We first compared the variation in splice-site strength of the splice-sites across both tissues through PCA analysis. The PCA analysis revealed that the within tissue/replicate variation is much lower in pollen compared to seedlings, which suggested that our ability to detect differential splicing would be higher in pollen compared to seedlings (Supplementary Figure 5B–D).

**Figure 4:**
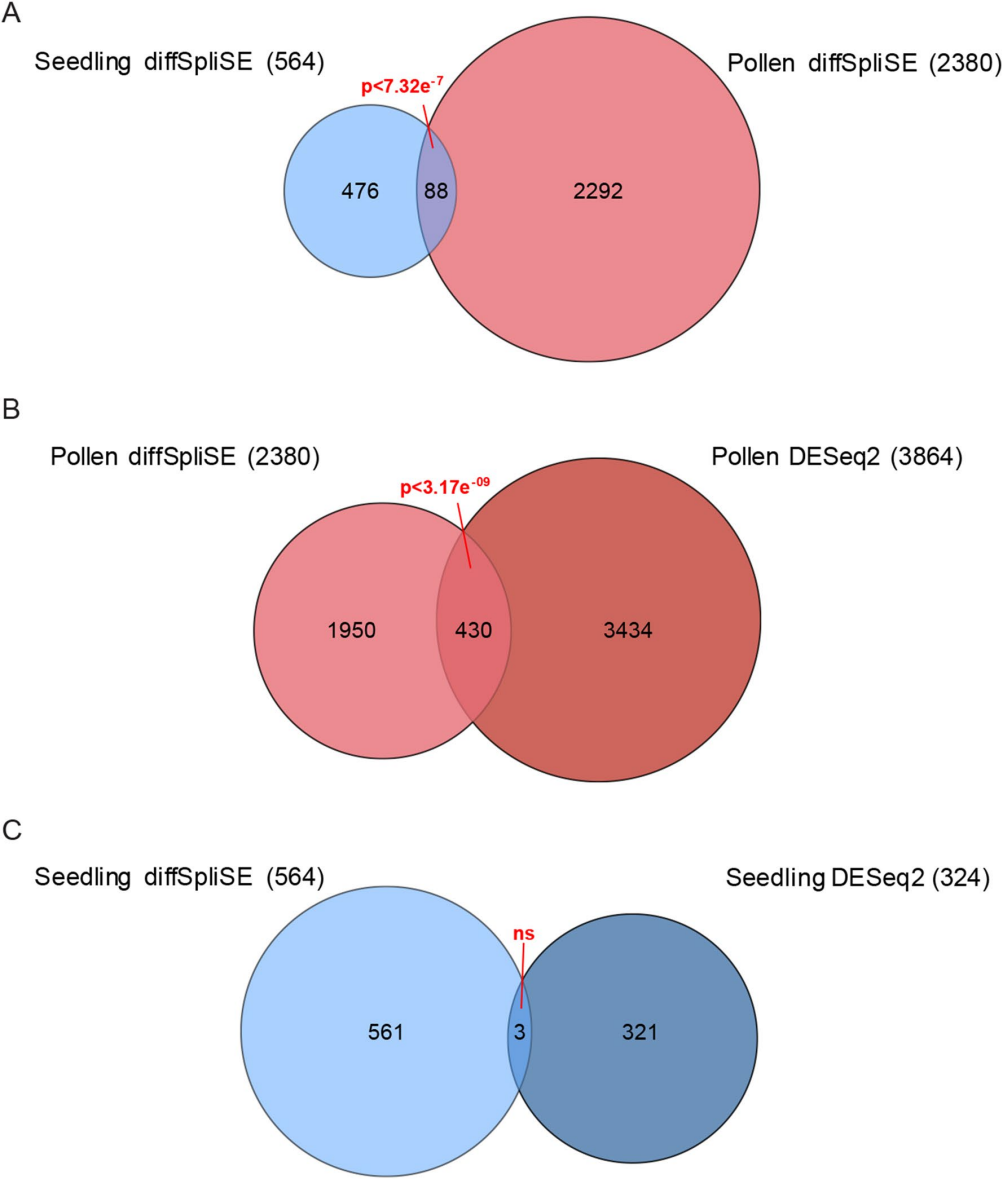
Down-regulation of *CWC15* in *cwc15-1* leads to global splicing defects and transcriptional differences. (**A**) Venn diagram showing number of genes differentially spliced in seedling and pollen tissue, between wild type and mutant. (**B**) Venn diagram comparing genes detected as differentially spliced and differentially expressed in pollen. (**C**) Venn diagram comparing genes detected as differentially spliced and differentially expressed in seedlings. Venn Diagram Plotter v1.5.5228 (https://omics.pnl.gov/software/venn-diagram-plotter) was used to generate Venn diagrams.

The analysis of differential splicing through diffSpliSE in SpliSER (Supplementary Dataset File 1) showed 620 splice sites to be differentially utilized in seedlings, corresponding to 564 genes. Most of the splice sites were canonical, consistent with the notion that CWC15 is an integral component of the core splicing machinery. We saw no clear bias in the prevalence of 5′ or 3′ splice site, 58%, and 42% respectively. SpliSER uses competition between splice sites as a parameter in assessing splice-site strength. The majority of differentially spliced sites (75%) had no competing splice sites observed in any sample, which indicates that they are constitutive splice sites undergoing intron retention (Supplementary Figure 4B). In pollen, we detected 3,997 splice sites to be differentially spliced, across 2,380 genes. 88 of these genes were common to both seedlings and pollen (Fig. 4A). Unlike in seedlings, where a vast majority of differentially-spliced sites showed a decrease in splice-site strength (99.7%), we saw an even distribution of up- and down-regulated splice sites in pollen, with no apparent bias towards a particular splicing event. Together these results suggest that the splicing defect observed in *cwc15-1* mutant seedlings is primarily a reduced capacity for the splicing of some introns (i.e. intron retention), rather than a change in splice-site preference (i.e. alternative usage of 3′ and 5′ splice sites, exon skipping, etc.).

Since differences in splicing can indeed lead to changes in expression levels^41,42^, we compared transcripts that were differentially up- or down-regulated (Supplementary Dataset File 1). 324 genes were differentially expressed more than twofold in seedlings, and 3,864 in pollen. We saw a significant overlap between differentially spliced and differentially expressed genes in pollen (Fig. 4B), but not in seedlings (Fig. 4C), suggesting that the *cwc15-1* splicing defects observed in seedlings may not be directly correlated with changes in gene expression. However, given that these results are derived from two RNA-seq replicates, we cannot rule out the tissue-specific differences observed in splicing and gene expression are due to differences in statistical power. To corroborate these results, we performed gene ontology (GO) enrichment analysis for differentially spliced and expressed genes in pollen and seedlings (Supplementary Dataset File 2). For differentially spliced genes in both seedlings and pollen we found enrichment for a wide range of processes including numerous GO terms involving metabolism and response to various stimuli with functions in both protein and nucleotide binding. This was also the case for differentially expressed genes in pollen. The number of enriched GO terms for differentially expressed genes in seedlings was considerably lower and we saw enrichment of response terms, the top three hits being related to iron homeostasis which do not appear in any of the other lists.

Taken together, these findings reveal three aspects of *CWC15* function. First, even a hypomorphic allele of *CWC15* leads to changes in splicing patterns. Second, compromising *CWC15* function has a direct and/or indirect effect on gene expression. Third, the effect of CWC15 differs between tissue types and/or stages of development.

### Loss of CWC15 function is female gametophytic and embryo lethal

The pleiotropic phenotypes in *cwc15-1* are caused by the downregulation of *CWC15* transcript levels, indicating that *cwc15-1* might be a hypomorphic allele. Therefore, we analyzed a T-DNA insertion allele, with the T-DNA residing in the third intron, termed *cwc15-2*. For this allele, we never recovered homozygous mutant progeny from heterozygous plants (*cwc15-2*^+*/*+^ n = 114, 48.3% vs. *cwc15-2*^+*/−*^ n = 122, 51.7%). When we opened siliques of selfed *cwc15-2*^+*/−*^ plants we found missing and shriveled ovules compared to WT, corresponding to aborted ovules (Supplementary Fig. 6). Reciprocal crosses of heterozygous *cwc15-2*^+*/−*^ and wild-type plants showed that transmission of the mutant allele via the female gametophyte was reduced from the expected 50% to 29% whereas the male gametophyte seemed not affected at all (Supplementary Table 3).

**Figure 5:**
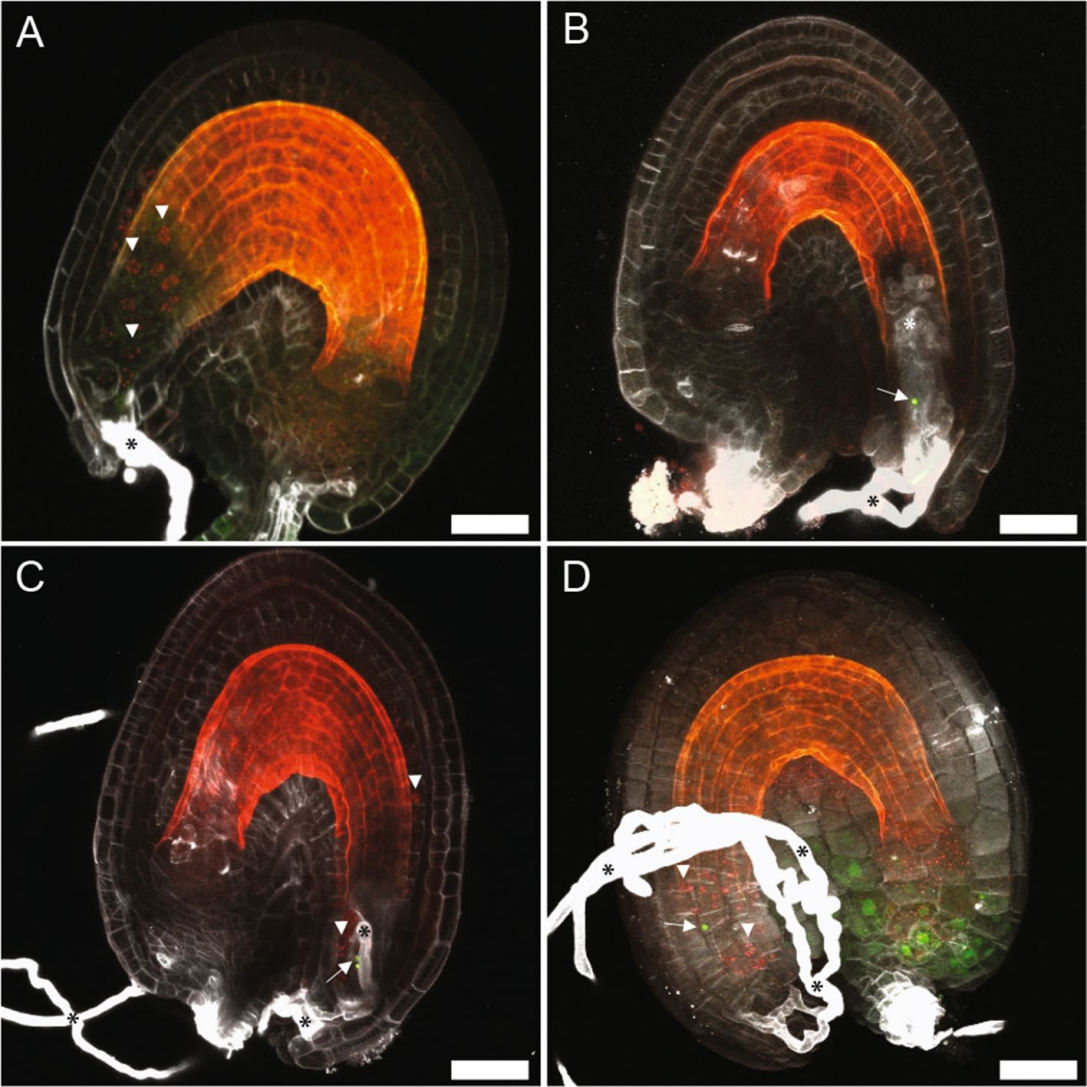
Loss of CWC15 function in *cwc15-2* leads to defects in double fertilization. (**A**–**D**) Overlay images of wild-type control (**A**) or CWC15 deficient ovules (**B**–**D**) 20 h after pollination expressing pollen double marker visualizing histones of fertilized endosperm nuclei in red (**A**,**C**,**D**), sperm nuclei in green (**B**,**C**,**D**), and SR2200 counter-staining of pollen tube cell walls in white (**A**–**D**). Arrowheads mark endosperm nuclei (red), arrows point to sperm nuclei (green), and asterisks indicate pollen tubes (white). Scale bar: 20 µm.

Next, we analyzed cell type-specific fluorescent marker lines for the female gametophyte expressed in the central cell, the synergid cells, or the egg cell. However, we could not detect any differences in *cwc15-2*^+*/−*^ compared to wild type in any of the marker crosses analyzed (n > 100), which suggested that cell identity was not affected in these lines (Supplementary Fig. 6, Supplementary Videos 1–3). This also suggests that the altered function of the female gametophyte rather than aborted development causes the observed decreased female transmission of *cwc15-2*^+*/−*^. To detect defects during pollen tube attraction and fertilization, we used a pollen multiple marker line. Sperm cell nuclei were labeled with a male gamete-specific Histone H3.3-YFP fusion protein (HTR10-YFP) and a centromeric CenH3-mCherry fusion protein (HTR12-mCherry). Upon fertilization, the HTR10-YFP protein is turned over while zygote and endosperm show mCherry fluorescence at the centromeric chromatin, resulting in distinct foci in the zygote and endosperm nuclei^43^. *CWC15* loss of function resulted in a range of phenotypic consequences in pollen tube perception and fertilization (Fig. 5). After successful double fertilization, endosperm nuclei in wild-type plants showed 15 spots of nuclear-localized mCherry signal and no YFP signal could be detected (Fig. 5A). In ovules of *cwc15-2*^+*/−*^ plants, however, we frequently observed pollen tubes without double fertilization as indicated by the absence of mCherry signal and the presence of nuclear YFP signal of unfused, persisting sperm cells (Fig. 5B). Also, we observed pollen tube overgrowth inside the ovule (Fig. 5B and C) as well as polytubey (Fig. 5D). Taken together, these observations suggest that CWC15 is required for efficient pollen tube reception and gamete interaction leading to successful double fertilization.

**Figure 6:**
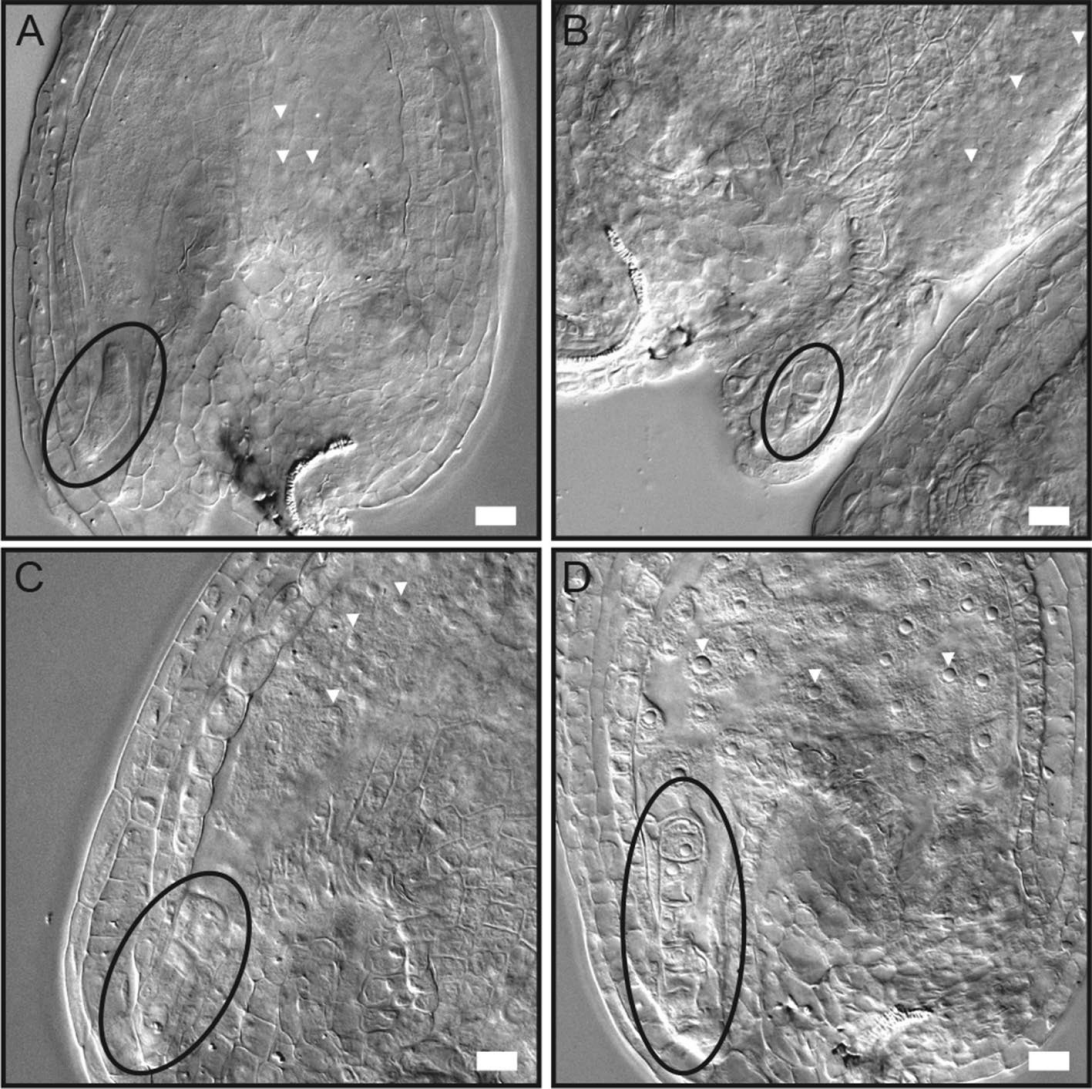
Zygote and embryo-arrest phenotypes in *cwc15-2*^+/−^ mutant embryos. (**A**) Embryo arrested at zygote stage. (**B**, **C**) Embryos arrested at 1-cell stage. (**D**) Embryo arrested at 2-cell stage. Zygote and embryos are encircled by ellipses. Arrowheads indicate nuclei of fertilized endosperm. Scale bar: 10 µm.

The ratio of aborted ovules from crosses of *cwc15-2*^+/−^ gynoecia pollinated with pollen from Col-0 anthers (13.6%, n = 132) indicated, as well as the aforementioned reciprocal crosses, that a low percentage of ovules in *cwc15-2*^+/*−*^ plants and their egg cells within can be fertilized. To investigate at which stage cwc15-2^−/−^ zygote/embryo development might be arrested, we looked at ovules in *cwc15-2*^+/−^ plants in self-pollinated flowers 72 h after pollination. We were able to identify seemingly aborted or delayed embryos at zygote and the earliest embryo stages of development (Fig. 6A-D). These results show that CWC15 function is important for female gametophyte development and fertilization and essential for embryogenesis, whereas the male gametophyte is not affected.

## Discussion

Although the eukaryotic spliceosome machinery is evolutionarily conserved, there are species-specific differences. The spliceosome and the activating NineTeen complex show differences in both the number and nature of proteins involved between yeast, human and Arabidopsis^26,44,45^. In Arabidopsis, for example, there seems to be a duplication and likely redundancy of factors playing major roles during splicing (e.g., Prp8 or Prp19)^33^. One of the single-copy genes previously associated with the splicing machinery is CWC15. Our thorough phylogenetic analysis indicated that CWC15 is present and conserved in virtually all eukaryotic genomes. Until recently, knowledge about the exact function of CWC15 has been scarce. Protein–protein interaction data revealed CWC15 as a protein associated with yeast and human spliceosomes^23,46^. CWC15 was later considered an integral part of the Prp19 complex/NTC through its direct interaction with CDC5^47,48^. In Arabidopsis, however, co-precipitation with Prp19 could not be demonstrated and CWC15 was therefore deemed not being part of the NTC^26,49^. In contrast, our mass spectrometry-based analysis showed co-precipitation of most Arabidopsis NTC components with CWC15, indicating that there is a close interaction of CWC15 with the NTC in Arabidopsis. We also detected other components of the core spliceosomal machinery, which is in line with recent structural data gained from yeast and human spliceosomes. The yeast homolog Cwf15/Cwc15 interacts with several U5 snRNA components and together with Prp45 is thought to be important for the stability of the spliceosomal core or main body^20,45,50,51,52^. Like the yeast multi-protein complex, the human CWC15 counterpart Ad-002 is also present at the core region of the human spliceosome^21,44,53^ and might be modified by human spliceosome-specific peptidyl-prolyl isomerases for functional catalytic activity^54^. Structures of plant spliceosomes have yet to be determined, but Arabidopsis CWC15 possibly has a similarly central role in the spliceosomal multi-protein complex as do the homologous proteins in yeast and human.

Alternative splicing (AS) is very common in humans, where essentially 100% of the transcripts have at least two isoforms^55^. The prevalent form of AS in animals is exon skipping^56^. We have utilized an approach that allows the detection of specific splice-sites that show aberrant usage between samples^40^. It has been estimated that 61% of Arabidopsis genes undergo AS, with the majority of AS events being intron retention^57^ which in turn leads to nonsense-mediated decay (NMD) of the affected transcript^56^. However, in plants retained introns do not always trigger NMD^58^. In Arabidopsis, AS can be achieved by cell type-specific expression patterns of splicing factors^59,60^ and this has been studied mainly during flowering time^61^. In our work, RNA sequencing analysis in a hypomorphic mutant background with seedling and adult growth defects showed that a decrease in CWC15 protein abundance causes clear splicing defects. While differences in splicing can lead to expression differences, in *cwc15* mutants, the observed differences in gene expression cannot be solely attributed to splicing defects. Additional changes in expression may be secondary effects resulting from splicing abnormalities. The phenotypic severity increased strongly in a putative knock-out mutant of CWC15 which showed pleiotropic fertilization defects and was embryo lethal. It has been previously shown that mutations in splicing factors primarily affect the viability of the female gametophyte^11,12,62,63^. Likewise, loss of the splicing factor CWC15 caused strongly decreased transmission via the female gametophyte while the pollen was entirely unaffected. Several studies detected new transcripts, differential splicing and even alternative transcriptional start sites in pollen when compared to leaf tissues^64,65^. However, transcripts enriched in pollen appear to have roles in splicing which could contribute to increased robustness of the pollen when compared to the egg cell. It has been shown that so-called housekeeping genes can be linked to specific mutant phenotypes as is the case for various splicing factors that affect the development of the female gametophyte^66^. This could explain why the loss of CWC15, and other splicing factors causes different phenotypes between male and female gametophytes. Curiously, recent research showed that a pair of splicing factors specifically affects the male gametophyte in double mutants but not the female gametophyte^67^. Future research will show if the composition of the splicing machinery in plants is indeed tissue-specific and is possibly involved in the different needs of various tissue types during development.

## Material and methods

### Plant material and in silico analysis

The wild-type plant line used was the Col-0 accession and plants were grown as previously described^35^. The T-DNA insertion line *cwc15-2*^+/−^ (SALK_010555, Col-0) was provided by the Nottingham Arabidopsis Stock Centre (NASC). The transgenic marker lines for specific cell types in the female gametophyte *pEC1:HTA6-3xeGFP*, *pNTA* > > *ntdTomato*, and *pMEA:3xGFP* as well as the multi-color marker were previously described^43,68^.

Acquisition of protein sequences, sequence alignment, and generation of the phylogenetic tree was performed as shown before^69^. Representation of protein sequence alignment in RasMol color and sequence conservation was done with CLC Genomics Workbench software version 10.1.1.

### Molecular cloning

The sequences of primers used in this study are listed in Supplementary Table 4. Both the CWC15 genomic rescue construct and the translational GFP fusion construct were generated by PCR amplifying a 2,825 bp fragment including 1,087 bp upstream of the CWC15 start codon and cloned into *GIIK-tNOS* (*CWC15* genomic start and stop primers) and *GIIK-3xeGFP-tNOS* (*CWC15* genomic start and -TAG stop primers), respectively, using restriction enzymes SalI/BclI. *GIIK-pRPS5A:CWC15-tNOS* was cloned by PCR amplifying the 693 bp CWC15 coding sequence (CDS) with CWC15 CDS start and stop primers and inserting the CDS into *GIIK-pRPS5A-tNOS*^70^ using restriction site BclI. The construct for early embryo expression was cloned by amplifying a 2 kb promoter element upstream of the *AT3G10100* start codon^35^ with *AT3G10100* start and stop primers and inserting the amplicon into *GIIK-tNOS*, using restriction enzymes XhoI/SmaI. The *CWC15* CDS was subsequently amplified with CWC15 CDS start and stop primers, and restriction enzyme BclI was used for cloning into *GIIK-pAT3G10100-tNOS*.

### Whole-genome sequencing

Genomic DNA was extracted from pooled *cwc15-1* seedlings 6 days after germination (6 dag), using the Qiagen DNeasy Plant Mini Kit. Libraries for DNA Next Generation Sequencing (NGS) were prepared with 1 µg DNA, using the Illumina TruSeq DNA PCR-free Low Throughput Library Prep Kit and Single Indexes Set A, and sequenced on an Illumina HiSeq 2000 machine. The transgenic insertion site in *cwc15-1* was initially determined by aligning sequencing reads to the Arabidopsis genome (https://www.araport.org/data/araport11) and the border region of the transgenic construct, using CLC Genomics Workbench software version 10.1.1. The insertion site was confirmed by PCR genotyping of border regions, using transgenic and genomic primers (LB and *cwc15-1* genotyping start primers, 341 bp; RB and *cwc15-1* genotyping stop primers, 323 bp), and subsequently by Sanger sequencing of PCR products.

### PCR genotyping

The *cwc15-2 *T-DNA allele was genotyped with primers *cwc15-2* RP, *cwc15-2* LP, and T-DNA-specific primer LBa1 (wild-type allele RP + LP 824 bp, T-DNA containing allele RP + LBa1 approximately 450 bp). The *cwc15-2*^−^ insertion site was determined by sequencing the T-DNA allele PCR product, using primer LBb1.3.

### sqRT-PCR and qRT-PCR

Total RNA was extracted from Col-0 and *cwc15-1* mature pollen or pooled seedlings 6 dag, using the Qiagen RNeasy Plant Mini Kit and on-column DNase digest (Qiagen RNase-free DNase Set). Reverse transcription was carried out with 1 µg total RNA using the RevertAid RT Reverse Transcription Kit (Thermo Scientific). For sqRT-PCR analysis, the following PCR conditions were used: 94° for 5 min followed by 30 or 35 cycles of 94° for 10 s, 58° for 30 s, and 72° for 1 min with a final extension step 72° for 5 min. For qRT-PCR analysis, we used the intronless and ubiquitously expressed control gene *UBQ10* for normalization and the following PCR program: 95° for 3 min followed by 40 cycles of 95° for 10 s, 60° for 10 s, and 72° for 20 s. Gene *AT3G08950* as an example for splice-site usage was randomly chosen from among the top sites from Supplementary Dataset 1. Example gene *AT2G34060* was among the not statistically significant sites. All primers used can be found in Supplementary Table 4.

### RNA sequencing, splicing, and GO enrichment analysis

Gene lists for differential expression and splicing analysis as well as GO terms can be found in the Supplementary Dataset Files 1 and 2. As described above, total RNA was extracted from two biological replicates for both pollen and seedlings and libraries for RNA NGS were prepared with 1 µg total RNA, using Illumina TruSeq RNA Library Prep Kit v2 and sequenced on an Illumina HiSeq 2000 machine. RNA-seq data were mapped using STAR v2.5.2^71^, taking only uniquely mapping reads, with minimum intron size 20, and maximum intron size 6,000. A splice junction BED file was generated using RegTools v0.5.2^72^ with the same intron limits. Each mapped RNA-seq sample was processed with SpliSER v0.1.1 and analyzed using the diffSpliSE pipeline^40^. To maintain the accuracy of the quantification, a splice site would be filtered out unless each replicate being assessed had at least 10 reads showing evidence of its utilization, or non-utilization. When comparing RNA from wild-type and *cwc15-1* seedlings, SpliSER detected 247,741 splice sites with sufficient read coverage in all samples; in pollen 66,191 splice sites were detected with sufficient read coverage in all samples.

For differential gene expression analysis, read counts were extracted from RNA-seq alignments using featureCounts v1.5.1^73^. Differential gene expression was called using DESeq2 v1.22.2^74^ with read counts normalized using the sizeFactors() function. Genes with a corrected p-value < 0.05 and log2FoldChange >  ± 2 were taken as differentially expressed. Differential gene expression PCA plots used DESeq2 regularized-log transformation read counts (rlog() function). Overlaps between gene lists were tested through hypergeometric probabilities. Venn diagrams were generated with Venn Diagram Plotter Software v1.5.5228 (https://omics.pnl.gov/software/venn-diagram-plotter). For gene ontology (GO) enrichment analysis, we took lists of genes that showed differential expression or that contained differentially spliced sites. Gene lists were uploaded to the AgriGO web portal (v2.0)^75^, and we performed singular enrichment analysis using the TAIR10_2017 background gene set. A corrected p-value less than 0.05 was considered to be significant.

### Microscopy

Images of seedlings and plants were taken with a Canon EOS 1000D camera. Clearing of ovules or embryos and staining with SR2200 were done as previously described^35,43^. Images of embryos were taken with a Zeiss Axio Imager. Fluorescent proteins were imaged using Leica TCS SP8, Olympus FV1000, or Zeiss LSM780 NLO confocal laser scanning microscopes and LAS X, FLUOVIEW, or ZEN software respectively. Images were processed using ImageJ version 1.52i and Adobe Photoshop and Illustrator CS6.

### Immunoprecipitation and LC–MS/MS analysis

Precipitation of GFP-tagged proteins from seedlings 6 dag and subsequent mass spectrometry analysis was in essence the exact same procedure as was described previously^76,77^. Briefly, 1–2 g fresh weight seedling material was ground in liquid nitrogen, using mortar and pestle. The resultant seedling powder was suspended in 2–3 ml lysis buffer (150 mM NaCl, 50 mM Tris pH 7.5, 2 mM EDTA, 0.5% Triton X-100) containing 20–30 µl Protease Inhibitor Cocktail (P9599, Sigma-Aldrich). After centrifugation, the supernatant was filtered with Miracloth (Calbiochem) and 2 ml of the supernatant was incubated with 20 µl GFP-Trap beads (Chromotek) for 3 h at 4 °C, using a tube rotator. The magnetic beads were washed three times with wash buffer (150 mM NaCl, 50 mM Tris pH 7.5, 0.1% Triton X-100) on a magnetic stand. Bead-bound proteins were eluted by boiling in 1 × Laemmli buffer and purified by SDS-PAGE followed by in-gel Trypsin digest. The digested peptides were subjected to LC–MS/MS analysis and MS spectra were processed with MaxQuant package software version 1.5.2.8 with integrated Andromeda search engine^78^.

## Supplementary information

Supplementary Dataset File 1.

Supplementary Dataset File 2.

Supplementary Legends.

Supplementary Figure 1.

Supplementary Figure 2.

Supplementary Figure 3.

Supplementary Figure 4.

Supplementary Figure 5.

Supplementary Figure 6.

Supplementary Figure 7.

Supplementary Video 1.

Supplementary Video 2.

Supplementary Video 3.

Supplementary Table 1.

Supplementary Table 2.

Supplementary Table 3.

Supplementary Table 4.
