# Supplementary material for: The integral spliceosomal component CWC15 is required for development in Arabidopsis
Source: Sci Rep. 2020 Aug 7;10:13336. doi: 10.1038/s41598-020-70324-3 (PMC7415139; doi:10.1038/s41598-020-70324-3)
Supplement: Supplementary file 3 — Supplementary Legends. [file 41598_2020_70324_MOESM3_ESM.docx]

**Supplementary Figure Legends**

**Supplementary Figure 1. Phylogeny of CWC15 proteins.**

A. Unrooted phylogenetic tree representing CWC15 homologs from various eukaryotic phyla. Radial tree layout with bootstrap support close to branching points. Species used for phylogenetic analysis are listed in Supplementary Table 1. Note the distance between plant and animal CWC15 groups. Image of phylogenetic tree was exported from MEGA software version 7.0.18 (http://www.megasoftware.net).

B. Alignment comparing several CWC15 homologs from plants and animals and additionally fungal CWC15. Individual amino acids are depicted in RasMol color scheme, degree of overall amino acid conservation is shown both as line plot and sequence logo. Image of alignment was exported from CLC Genomics Workbench software version 10.1.1 (https://digitalinsights.qiagen.com/products-overview/discovery-insights-portfolio/analysis-and-visualization/qiagen-clc-genomics-workbench/).

**Supplementary Figure 2. Expression pattern of *CWC15* genomic construct in male gametophyte, embryo, and seedling.**

A-C. Both vegetative and generative nuclei show CWC15-3xGFP signal during male gametophyte development in unicellular microspore (A), bicellular pollen (B), and tricellular pollen (C). Inset (B-D): DAPI stained nuclei in male gametophytes.

D-J. CWC15-3xGFP is present in all nuclei throughout embryo development at 1-cell (D), 4-cell (E), early globular (F), triangular (G), late-heart (H), torpedo (I), and bent cotyledon (J) stages.

K. *gCWC15-3xGFP* expression in epidermal cells. The image is a maximum projection of z-stacks across abaxial cotyledon epidermal cells. Nuclear-localized CWC15-3xGFP is shown in green, cell outlines are stained with propidium iodide (magenta).

L. *gCWC15-3xGFP* expression in seedling shoot. Nuclear-localized CWC15-3xGFP in primary leaves of a 7-day-old seedling is shown in green, autofluorescence is shown in red.

Scale bar: (A-I and K) 5 µm, (J and L) 100 µm.

**Supplementary Figure 3. sqRT-PCR of gene products close to genomic insertion site.**

CWC15 transcript is less abundant in the *cwc15-1* background due to insertion in the promoter region. Gene names or loci numbers are depicted above the corresponding lanes of the gel image, lanes 1-4 from left to right for each gene transcript tested correspond to 1) Col-0 DNA, 2) H2O, 3) *cwc15-1* cDNA, and 4) Col-0 cDNA. Note that PCR probes for tested genes and corresponding control *Actin 2* were run on separate DNA gels. *Actin 2* sqRT-PCR was done with 30 cycles, whereas PCRs for *AT3g13190*, *CWC15*, and *AT3g13210* ran with 35 cycles. Multiple exposures are shown, overexposure of DNA gel images is due to weakness of some of the cDNA PCR products.

**Supplementary Figure 4. qRT-PCR showing *cwc15-1* hypomorphic background and example of splice-site usage.**

A. *CWC15* is strongly down-regulated in hypomorphic *cwc15-1* mutant background.

B. Example of less utilized splice site in *AT3G08950* gene leading to intron retention compared to *AT2G34060* gene showing no significant difference in splice-site usage. Primers used for splice-site usage examples are spanning respective splice sites and binding sites are in exon and intron.

For qPCR analysis, two separate pools of seedling RNA (biological replicates) were used for each wild type and mutant. Fold change was calculated with log2 -∆CT method and first wild type biological replicate was set to one. Sample reactions were pipetted as three technical replicates and according error bars show standard deviation.

All bar graphs were created with Microsoft Excel 2016.

**Supplementary Figure 5. PCA plot analysis.**

A. Gene expression (regularized log-transformed read counts).

B. Splice-site strength (SSE) for splice-sites with 10-reads across all RNA-seq samples (33,312 sites).

C. SSE for splice-sites with 10-reads across all seedling samples (242,741 sites).

D. SSE for splice-sites with 10-reads across all pollen samples (66,191 sites).

Images of PCA plots were generated with R version 4.0.2 (https://www.r-project.org/).

**Supplementary Figure 6. Loss of CWC15 function leads to decreased fertility.**

Only a few ovules abort in siliques of WT plants (1.53 %, n = 393). This percentage is highly increased in siliques of heterozygous *cwc15-2+/-* plants (20.9 %, n = 392).

Bar graph was created with Microsoft Excel 2016.

**Supplementary Figure 7. Expression of cell type-specific markers is not disturbed in the female gametophyte.**

A and D. Cell type-specific expression of *pEC1:HTA6-3xeGFP* in *cwc15-2+/-* mutant background.

B and E. Cell type-specific expression of *pNTA>>ntdTomato* in *cwc15-2+/-* mutant background.

C and F. Cell type-specific expression of *pMEA:3xeGFP* in *cwc15-2+/-* mutant background.

Close-up images of cell-specific nuclear expression in (D-F) compared to overview images in (A-C).

eGFP signal in green (A, C, D, F), autofluorescence in red (D), SR2200 counter-staining of cell walls in white (A-E), and tdTomato signal in red (B and E). DIC/fluorescence overlay image is shown in (F).

Arrowheads in (A and D) point to egg cell nucleus, arrows in (B and E) mark synergid nuclei, and asterisks in (C and F) label central cell nucleus. Apart from the nucleus, note that GFP fluorescence in (C and F) is also visible in the central cell cytoplasm. Scale bar: 20 µm.

**Supplementary Video Legends**

**Supplementary Video 1. 3D project movie of corresponding picture in Supplementary Figure 7D showing close-up of egg cell marker expression.**

**Supplementary Video 2. 3D project movie of corresponding picture in Supplementary Figure 7E showing close-up of synergid cell marker expression.**

**Supplementary Video 3. 3D project movie of corresponding picture in Supplementary Figure 7D showing close-up of central cell marker expression.**

**Supplementary Table Legends**

**Supplementary Table 1. Species used for phylogenetic analyses.**

**Supplementary Table 2. CWC15-associated proteins identified by mass spectrometry.**

Overview of co-immunoprecipitated proteins predicted or experimentally shown to be involved in spliceosomal activity. In line with recent structural data from human activated spliceosomes, the Arabidopsis loci and their corresponding protein IDs are listed next to their human counterparts. MS data are delineated by protein sequence coverage, Andromeda identification score for MS/MS spectrum, signal intensity, and MS/MS spectral counts.

**Supplementary Table 3. Reciprocal cross of *cwc15-2^+/-^* with Col-0.**

Whereas transmission of the mutant allele via the pollen is not disturbed, transmission via the female gametophyte is strongly affected by the loss of functional CWC15.

**Supplementary Table 4. List of primers used in this study.**
