# Supplementary figures and images for: The integral spliceosomal component CWC15 is required for development in Arabidopsis
Source: Sci Rep. 2020 Aug 7;10:13336. doi: 10.1038/s41598-020-70324-3 (PMC7415139; doi:10.1038/s41598-020-70324-3)

H. andi caliginosa

## Plants

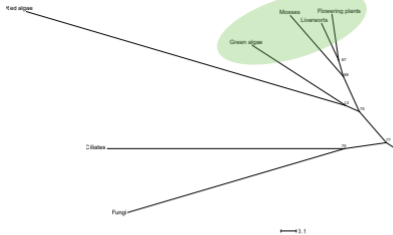

## Animals

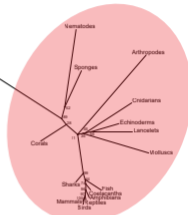

B

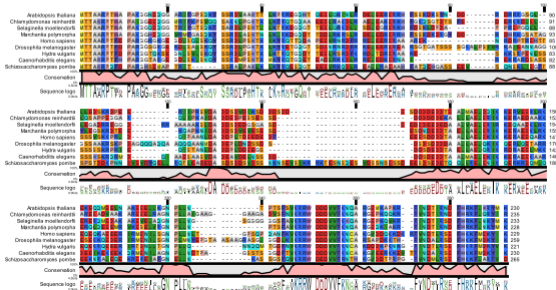

Supplement: Supplementary file 4 — Supplementary Figure 1. [file 41598_2020_70324_MOESM4_ESM.pdf]

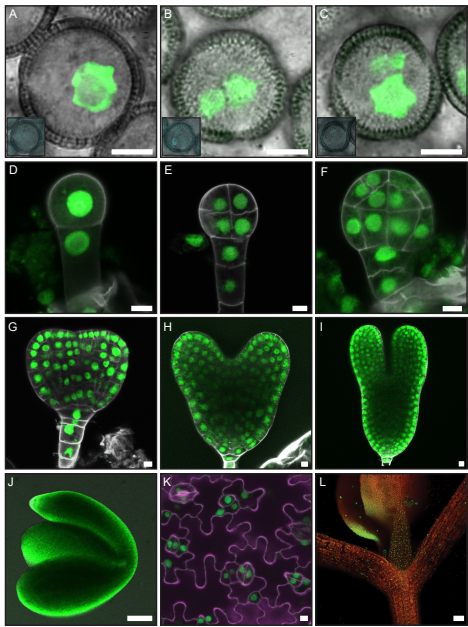

Supplement: Supplementary file 5 — Supplementary Figure 2. [file 41598_2020_70324_MOESM5_ESM.pdf]

*Actin 2*

*AT3G13190*

*CWC15*

*AT3G13210*

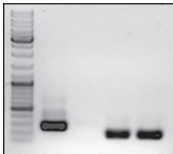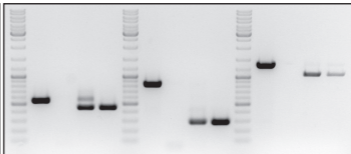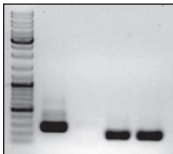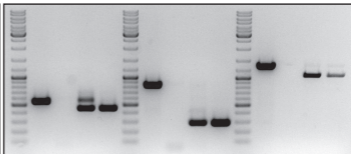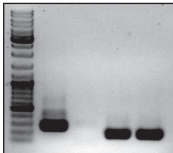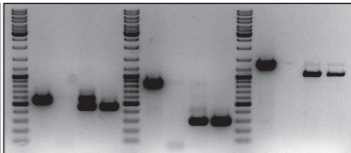

Supplement: Supplementary file 6 — Supplementary Figure 3. [file 41598_2020_70324_MOESM6_ESM.pdf]

**A*****CWC15***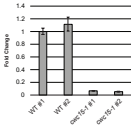**B*****AT3G08950***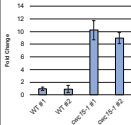***AT2G34060***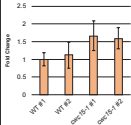

Supplement: Supplementary file 7 — Supplementary Figure 4. [file 41598_2020_70324_MOESM7_ESM.pdf]

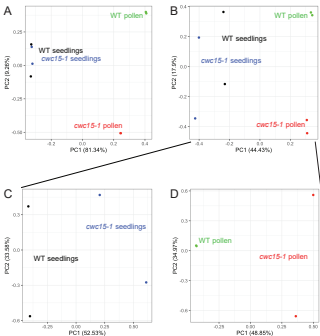

Supplement: Supplementary file 8 — Supplementary Figure 5. [file 41598_2020_70324_MOESM8_ESM.pdf]

% aborted oocytes

25  
20  
15  
10  
5  
0

WT

*owc15-2*

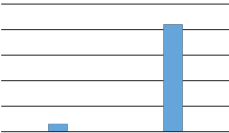

Supplement: Supplementary file 9 — Supplementary Figure 6. [file 41598_2020_70324_MOESM9_ESM.pdf]

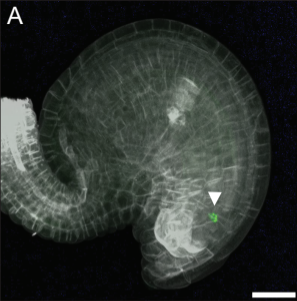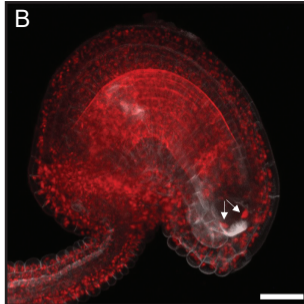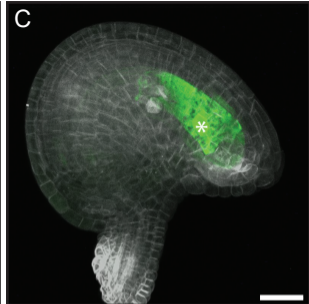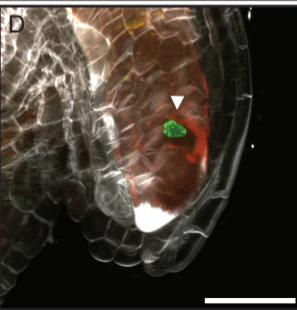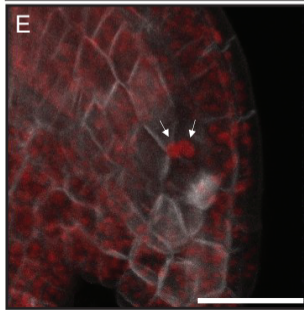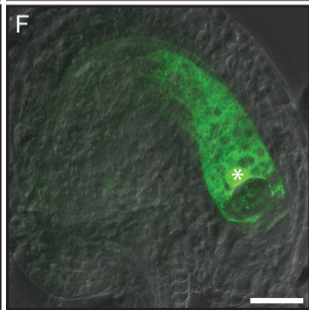

Supplement: Supplementary file 10 — Supplementary Figure 7. [file 41598_2020_70324_MOESM10_ESM.pdf]
