# Supplementary material for: The integral spliceosomal component CWC15 is required for development in Arabidopsis
Source: Sci Rep. 2020 Aug 7;10:13336. doi: 10.1038/s41598-020-70324-3 (PMC7415139; doi:10.1038/s41598-020-70324-3)
Supplement: Supplementary file 14 — Supplementary Table 1. [file 41598_2020_70324_MOESM14_ESM.pdf]

Phylum	Species name
Flowering plants	*Arabidopsis thaliana*
Liverworts	*Marchantia polymorpha*
Mosses	*Physcomitrella patens*
Green algae	*Chlamydomonas reinhardtii*
Red algae	*Chondrus crispus*
Ciliates	*Stylonychia lemnae*
Fungi	*Schizosaccharomyces pombe*
Sponges	*Amphimedon queenslandica*
Nematodes	*Caenorhabditis elegans*
Corals	*Acropora digitifera*
Molluscs	*Octopus bimaculoides*
Cnidarians	*Hydra vulgaris*
Lancelets	*Branchiostoma belcheri*
Echinoderms	*Strongylocentrotus purpuratus*
Arthropods	*Drosophila melanogaster*
Sharks	*Callorhinchus milii*
Fish	*Danio rerio*
Coelacanths	*Latimeria chalumnae*
Amphibians	*Xenopus tropicalis*
Reptiles	*Anolis carolinensis*
Birds	*Fulmarus glacialis*
Mammals	*Homo sapiens*
