# Supplementary material for: The integral spliceosomal component CWC15 is required for development in Arabidopsis
Source: Sci Rep. 2020 Aug 7;10:13336. doi: 10.1038/s41598-020-70324-3 (PMC7415139; doi:10.1038/s41598-020-70324-3)
Supplement: Supplementary file 15 — Supplementary Table 2. [file 41598_2020_70324_MOESM15_ESM.pdf]

Homo sapiens	*Arabidopsis thaliana*	AGI locus	UniProt ID	Peptides	Sequence coverage [%]	Score	Intensity	MS/MS Count
**U5 snRNP**								
Brr2	BRR2/MAC14/EMB1507	*AT1G20960*	O48534	29	14,6	79,507	444470000	43
U5-40	MAC17	*AT2G43770*	O22826	5	17,8	8,6494	59862000	5
Prp8	PRP8B	*AT4G38780*	Q9SSD2	23	9,4	58,79	292690000	31
Snu114	GFA1/MEE5/CLO	*AT1G06220*	Q9LNC5	14	17,7	64,336	266600000	19
U5 sm ring								
Sm B/B'		*AT5G44500*	Q9FI15	2	5,9	3,1189	14232000	2
Sm D2		*AT3G62840*	Q8RUH0					
		AT2G47640	Q8RUH0					
Sm D1	SmD1a	*AT3G07590*	Q9SSF1					
	SmD1b	*AT4G02840*	Q9SY09					
Sm D3		*AT1G20580*	Q9LM92					
Sm E		*AT2G18740*	Q9ZV45	1	14,1	10,073	10818000	1
Sm F	RUXF	*AT4G30220*	F4JPK5					
Sm G		*AT2G23930*	O82221	1	22,4	3,2787	5851700	1
		AT3G11500	Q9CAX7					
**NTC**								
Cdc5	CDC5/MAC1	*AT1G09770*	P92948	18	21,6	39,897	228190000	19
Prp19	MAC3A	*AT1G04510*	F4I5P3	12	27	32,736	130360000	10
	MAC3B	*AT2G33340*	O22785	12	28,8	57,663	448850000	14
Spf27	MOS4	*AT3G18165*	Q949S9	1	4,3	3,2353	17435000	1
Syf1	MAC9	*AT5G28740*	Q9LKU3	14	15,9	33,249	171140000	14
Syf2		*AT2G16860*	Q9ZVX7					
Syf3	MAC10	*AT5G41770*	F4JZX8	13	16,3	16,361	138040000	11
Isy1	MAC1	*AT3G18790*	Q9LS97	1	3,7	1,3055	3306300	1
CTNNBL1		*AT3G02710*	A3KPF3					
HSP73	HSP70	*AT3G09440*	O65719	21	32,7	33,405	111960000	13
**NTR**								
RBM22	MAC5A	*AT1G07360*	Q9LNV5	6	12,5	26,372	42295000	6
SKIP	SKIP	*AT1G77180*	O80653	8	15,3	30,515	91681000	8
G10		*AT4G21110*	O49553	1	11	14,075	4845200	1
PPIL1		*AT2G36130*	Q9SIH1					
Ad-002	CWC15	*AT3G13200*	Q9LK52	26	61,3	304,66	18999000000	56
PRL1	PRL1	*AT4G15900*	Q42384	6	15,8	18,683	43612000	7
Aquarius	EMB2765	*AT2G38770*	Q8L5Y4	12	8,9	28,811	122810000	12
**Splicing Factor**								
SRRM2/SRm300		*AT3G49601*	B3H6J5					
Cwc22		*AT1G80930*	Q9SAG7	1	1,2	1,4812	1191100	1
RNF113A		*AT5G06420*	Q9FNG6					
		AT1G01350	Q8GX84					
Prp2	EMB2733/ESP3	*AT1G32490*	Q8VY00					
Prp17		*AT1G10580*	A4FVN8	4	7,9	9,6605	34635000	4
