# Supplementary material for: The integral spliceosomal component CWC15 is required for development in Arabidopsis
Source: Sci Rep. 2020 Aug 7;10:13336. doi: 10.1038/s41598-020-70324-3 (PMC7415139; doi:10.1038/s41598-020-70324-3)
Supplement: Supplementary file 16 — Supplementary Table 3. [file 41598_2020_70324_MOESM16_ESM.pdf]

Parental genotype		F1 genotype		
♀	♂	het % (n)	WT % (n)	Σ
***cwc15-2**$^{+/-}$*	**WT**	29.2 (78)	70.8 (189)	267
**WT**	***cwc15-2**$^{+/-}$*	48.3 (73)	51.7 (78)	151
