# Supplementary material for: The integral spliceosomal component CWC15 is required for development in Arabidopsis
Source: Sci Rep. 2020 Aug 7;10:13336. doi: 10.1038/s41598-020-70324-3 (PMC7415139; doi:10.1038/s41598-020-70324-3)
Supplement: Supplementary file 17 — Supplementary Table 4. [file 41598_2020_70324_MOESM17_ESM.pdf]

cwc15-1 genotyping start	5'-TTCGGACTCTAGCTAGAGTCAAGCA-3'
cwc15-1 genotyping stop	5'-ATGGACGAGCTGTACAAGTAAAGAT-3'
LB	5'-CTGAGAGTTCTCGTCGTCCTTATCT-3'
RB	5'-TGCTGCAGTCGTCATCTTCAGAAAA-3'
cwc15-2 RP	5'-AGATACTTAGAGAAGCGCGAGAGAG-3'
cwc15-2 LP	5'-GTGCTCTCCACATCTTCAGTTACCA-3'
Lba1	5'-GTGCTCTCCACATCTTCAGTTACCA-3'
LBb1.3	5'-ATTTTGCCGATTTCGGAAC-3'
CWC15 genomic start	5'-TATAGTCGACTAAGAGTTGTGACTTCAGAGCTAG-3'
CWC15 genomic stop	5'-TATATGATCATCACTTCATGTATCTATGCAGGA-3'
CWC15 genomic -TGA stop	5'-TATATGATCATCACTTCATGTATCTATGCAGGA-3'
CWC15 CDS start	5'-TATATGATCAATGACGACTGCAGCACGACCAACCT-3'
CWC15 CDS stop	5'-TATATGATCATCACTTCATGTATCTATGCAGGA-3'
p*AT3G10100* start	5'-TATACTCGAGGGTTTCGTGAGAGAGGACTT-3'
p*AT3G10100* stop	5'-TATACCCGGGGGTTTCGTGAGAGAGGACT-3'
ACTIN 2 (*AT3G18780*) sqRT start	5'-TGAGCAAAGAAATCACAGCACTTGC-3'
ACTIN 2 (*AT3G18780*) sqRT stop	5'-TCTGTGAACGATTCCTGGACCTG-3'
AT3G13190 sqRT start	5'-CTTTGACGCTGGTATTTCTGGAGGA-3'
AT3G13190 sqRT stop	5'-CTTCAGATCTTCAATGGCTTGATGA-3'
CWC15 (*AT3G13200*) sqRT start	5'-ATCTCAGAAGTATTCGTCCCGTGAT-3'
CWC15 (*AT3G13200*) sqRT stop	5'-CGTCACTTTTGATATCAACATCAG-3'
AT3G13210 sqRT start	5'-AGATACTTAGAGAAGCGCGAGAGAG-3'
AT3G13210 sqRT stop	5'-GTGCTCTCCACATCTTCAGTTACCA-3'
UBQ10 (*AT4G05320*) qPCR s	5'-GGCCTTGTATAATCCCTGATGAATAAG-3'
UBQ10 (*AT4G05320*) qPCR as	5'-AAAGAGATAACAGGAACGGAAACATAGT -3'
CWC15 (*AT3G13200*) qPCR s	5'-CAGATGGAAGAGCTAAACGC-3'
CWC15 (*AT3G13200*) qPCR as	5'-ACATCATCATCCCACCTCC-3'
AT3G08950 qPCR s	5'-GTCCCGATATCTGTCCTGACG-3'
AT3G08950 qPCR as	5'-CCTTAGAGGAAAAATCTCCAAATGT-3'
AT2G34060 qPCR s	5'-GAAACTCTCGTCGGTTCCGT-3'
AT2G34060 qPCR as	5'-TATGCTATAGTGCGCTGGCA-3'
